# Supplementary material for: Effects of Cymatocarpus solearis (Trematoda: Brachycoeliidae) on its second intermediate host, the Caribbean spiny lobster Panulirus argus
Source: PLoS One. 2023 Sep 29;18(9):e0287097. doi: 10.1371/journal.pone.0287097 (PMC10540948; doi:10.1371/journal.pone.0287097)
Supplement: S1 Table — Means and [95% confidence intervals] of all variables compared among four groups of lobsters categorized by the presence and intensity of infection by C. solearis (number of metacercarial cysts). (DOCX) [file pone.0287097.s001.docx]

**S1 Table. Response variables of *Panulirus argus* lobsters in four grades of infection by *Cymatocarpus solearis***

|  | **Uninfected** | **Lightly infected** | **Moderately infected** | **Heavily infected** |
| --- | --- | --- | --- | --- |
| **Response variables** | **(0 cysts)** | **(1-10 cysts)** | **(11-30 cysts)** | **(>30 cysts)** |
| ***Escape response*** |  |  |  |  |
| Delay to escape (s) | 0.91 [0.69, 1.13] | 0.72 [0.48, 0.96] | 0.58 [0.37, 0.80] | 0.91 [0.67, 1.14] |
| Duration of swimming bout (s) | 2.34 [1.67, 3.02] | 1.82 [1.13, 2.51] | 2.12 [1.48, 2.76] | 2.40 [1.71, 3.09] |
| Distance swum per bout (m) | 1.62 [1.03, 2.21] | 1.49 [0.89, 2.09] | 1.46 [0.90, 2.00] | 1.99 [1.39, 2.59] |
| Velocity (m/s) | 0.75 [0.62, 0.89] | 0.80 [0.66, 0.94] | 0.68 [0.56, 0.81] | 0.86 [0.72, 1.00] |
| Acceleration (m/s/s) | 0.55 [0.32, 0.79] | 0.58 [0.34, 0.82] | 0.53 [0.30, 0.75] | 0.66 [0.41, 0.90] |
| Force (N) | 0.36 [0.22, 0.50] | 0.37 [0.22, 0.52] | 0.34 [0.21, 0.48] | 0.42 [0.27, 0.57] |
| Work (J) | 0.43 [0.26, 0.60] | 0.50 [0.33, 0.67] | 0.36 [0.21, 0.52] | 0.56 [0.39, 0.74] |
| ***Metabolites in hemolymph*** |  |  |  |  |
| Albumin (mg/ml) | 3.50 [2.40, 4.60] | 4.35 [3.17, 5.54] | 4.08 [2.59, 5.57] | 3.69 [2.36, 5.02] |
| Glucose (mg/ml) | 0.23 [0.19, 0.27] | 0.19 [0.15, 0.23] | 0.25 [0.20, 0.30] | 0.17 [0.12, 0.21] |
| Cholesterol (mg/ml) | 3.94 [3.25, 4.64] | 4.55 [3.80, 5.31] | 4.28 [3.33, 5.23] | 3.79 [2.94, 4.63] |
| Total protein (mg(ml) | 97.4 [80.8, 113.9] | 100.9 [83.1, 118.7] | 108.5 [86.1, 130.9] | 86.9 [66.8, 106.9] |
| ***Neuromodulators*** |  |  |  |  |
| Dopamine (ng/ml) | 745.3 [420.7, 1069.9] | 526.9 [168.1, 885.7] | 494.5 [55.0, 933.9] | 329.5 [77.4, 736.3] |
| Serotonin (ng/ml) | 62.7 [43.8, 81.5] | 77.9 [59.9, 95.8] | 121.6 [102.7, 140.4] | 127.0 [108.6, 145.4] |
| ***Other variables*** |  |  |  |  |
| No. hemocytes/ml (x 10^6^) | 3.15 [2.45, 3.85] | 3.40 [2.75, 4.05] | 3.61 [2.96, 4.26] | 3.29 [2.66, 3.93] |
| Hepatosomatic index | 1.98 [1.82, 2.14] | 2.07 [1.90, 2.23] | 1.96 [1.77, 2.14] | 1.92 [1.74, 2.10] |
| Growth rate (mm CL/week) | 0.16 [0.11, 0.21] | 0.17 [0.12, 0.21] | 0.16 [0.11, 0.21] | 0.11 [0.06, 0.16] |

Means and [95% confidence intervals] of all variables compared among four groups of lobsters categorized by the presence and intensity of infection by *C. solearis* (number of metacercarial cysts)
