# Supplementary material for: Effects of Cymatocarpus solearis (Trematoda: Brachycoeliidae) on its second intermediate host, the Caribbean spiny lobster Panulirus argus
Source: PLoS One. 2023 Sep 29;18(9):e0287097. doi: 10.1371/journal.pone.0287097 (PMC10540948; doi:10.1371/journal.pone.0287097)
Supplement: Attachment — Submitted filename: Review of PONE-D-23-02958.docx [file pone.0287097.s003.docx]

**Manuscript Number:** PONE-D-23-02958
**Manuscript Title:** Effects of *Cymatocarpus solearis* (Trematoda: Brachycoeliidae) on its second intermediate host, the Caribbean spiny lobster *Panulirus argus*

Digenean trematode life cycles are fascinating and wonderfully complex. Many interesting ecological questions can be tested at the intermediate host-stage, as this is where behavioral manipulation of hosts is theorized to occur. In this paper, the authors quantified the effects of *C. solearis* infection on the Caribbean spiny lobster *P. argus*, a second-intermediate host for the parasite. There were clear differences in biomarkers of infection (e.g., concentrations of glucose and serotonin) and physical performance indicators (e.g., escape response) as a function of infection intensity in *P. argus*, findings that increase the likelihood of trophic transmission to an appropriate definitive host.

I believe the manuscript meets all PLOS ONE publication criteria. The work is scientifically sound, and I have no concerns about the experimental design. However, I would suggest that the authors implement some additional statistical tests to take full advantage of the data that they have painstakingly collected. For the most part, the authors limited themselves to using non-parametric hypothesis tests (Kruskal-Wallis) to test for significant differences. These tests can be useful, but they must be interpreted carefully because of problems with statistical vs. biological significance (i.e., Johnson 1995). More to the point, these tests have no real predictive abilities. Given the wealth of data collected for this project, I think the manuscript could be improved using glms and/or mixed models. These analyses would address the question: which variable(s) account for most of the variation in infection intensity? As a reader, I was left wondering about this. I have more detailed suggestions along these lines in the Methods/Results section of this review.

- Johnson, D. H. (1995). Statistical sirens: the allure of nonparametrics. *Ecology*, *76*(6), 1998-2000.

**Introduction**

Lines 79-89 -- why is visual assessment important? I think some additional context here would help. Is it just for researchers interested in the presence of the parasite, or do fishermen care as well? If appearance is important, this may be something to return to in the Discussion (beginning around line 460).

Lines 89-90 – the transition to the next paragraph about trophic transmission seemed a little abrupt. Perhaps another sentence is needed here?

Lines 98-102 – The ideas here seemed a little vague. I think the reader may be left wondering why these parasites have more consequences for intermediate hosts. The intermediate host needs to be eaten for the parasite to complete its lifecycle in a definitive host. Perhaps this should be stated? Also, how would parasites affect the lobster fishery?

**Methods/Results**

Line 182 – consider citing (and perhaps later discussing?) Shaw et al. 2009:

- Shaw, J. C., Korzan, W. J., Carpenter, R. E., Kuris, A. M., Lafferty, K. D., Summers, C. H., & Øverli, Ø. (2009). Parasite manipulation of brain monoamines in California killifish (Fundulus parvipinnis) by the trematode Euhaplorchis californiensis. *Proceedings of the Royal Society B: Biological Sciences*, *276* (1659), 1137-1146.

Suggestions for additional post-hoc testing:

It is possible to have a low p-value but a small effect size. In other words, the effect detected by the test that seemed so important (i.e., with the low p-value) is actually quite small. Cumming (2012) gives an excellent overview of this issue. To provide some additional context for your results from the Kruskal-Wallis tests, I might suggest testing the magnitude of effect for each significant p-value. The package “rstatix” is quite useful for basic statistical tests – I think Cohen’s D is preferred for parametric data and Wilcoxon effect size tests for non-parametric data.

# Cumming G. 2012. Understanding The New Statistics: Effect Sizes, Confidence Intervals, and Meta-Analysis (Multivariate Applications Series)

Suggestions for model building using information theoretic approaches:

You probably have your own methods, but in the interests of being as helpful as possible I’ll share with you what packages I typically use for multimodel inference in R.

- glmmTMB – package for building and testing generalized linear mixed models that has a great suite of diagnostic tools for assessing model fit:

<https://cran.r-project.org/web/packages/glmmTMB/index.html>

- DHARMa – add-on package with helpful tests for assisting with model convergence issues:

<https://cran.r-project.org/web/packages/DHARMa/vignettes/DHARMa.html>

- AICcmodavg – package for ranking and comparing models using information theoretic approaches:

<https://cran.r-project.org/web/packages/AICcmodavg/index.html>

You could bin the data into infected (1 or more cysts) and uninfected (0 cysts) categories. The goal here would be to rank and compare models testing the likelihood of infection. What variable(s) best predict whether a lobster is infected or not? Your response variable would be infection status (yes or no), a binary outcome (1 or 0), and you would fit the data to a binomial error distribution. In models that incorporate swimming performance as a predictor, you could consider adding trial number as a random effect as this might account for some of the variation in your response variable but would not be of biological/ecological importance.

You could also build models using infection intensity as your response variable (0 cysts; 1-10 cysts; 11-30 cysts; > 30 cysts). What variable(s) predict the likelihood of infection intensity in each case? You would fit the data to a negative binomial distribution if they showed evidence of overdispersion, which may be the case for the final category – otherwise, a Poisson would probably fit the data better. For variables that had multiple trials, consider using trial number as a random effect in your model structure.

Some thoughts on lobster size as a predictor variable:

I think you could make an argument for using lobster size as a fixed effect (predictor) in your models, or as a random effect since there doesn’t seem to be a clear linear relationship between size and infection status (lines 280-281). Admittedly, this result is a little surprising since larger (older) organisms tend to have more cysts, which you mention (lines 248-249). However, it may also be possible for crustaceans to “clear” their cysts over time. Older cysts can appear brown and withered (Blakeslee et al. 2020), a result of the long-term crustacean innate immune response (Lee and Soderhall 2002); however, you did not find evidence of melanization (line 472). One other intriguing possibility for this non-linear relationship is that larger adults are foraging in different areas compared to smaller individuals and so are not in proximity as often to the first-intermediate host. I have some unpublished data from stone crabs *Menippe mercanaria* that support this idea. Smaller individuals (up to approx. 40 mm carapace width) are sympatric with Panopeid mudcrabs in shallow water oyster reefs. These individuals often have trematode cysts. Larger stone crabs, on the other hand, tend to burrow more and seek out deeper habitats, meaning that they are not as close on average to the first-intermediate host, the mudsnail *Ilyanassa obsoleta.* I rarely find cysts in these larger stone crabs.

All in all, since the biological relationship between size and infection status seems unclear, I might argue for using size as a random effect since it is not an important predictor of infection but may still account for some variation in your response variables.

- Blakeslee, A., Ruocchio, M., & Moore, C. S. (2020). Altered susceptibility to trematode infection in native versus introduced populations of the European green crab.
- Lee, S. Y., & Söderhäll, K. (2002). Early events in crustacean innate immunity. *Fish & shellfish immunology*, *12*(5), 421-437.

**Discussion**

I’ll mostly hold off on commenting on this section for now, as your results may change if you decide to incorporate my suggestions for additional analyses. Ultimately, I think what you have is great, but I think you could do a lot more with your data.

One note on the serotonin (5-HT) results:

Would elevated levels of 5-HT affect lobsters’ ability, or interest, to shelter together in casitas? How would this affect the fishery?
