# Supplementary material for: Effects of Cymatocarpus solearis (Trematoda: Brachycoeliidae) on its second intermediate host, the Caribbean spiny lobster Panulirus argus
Source: PLoS One. 2023 Sep 29;18(9):e0287097. doi: 10.1371/journal.pone.0287097 (PMC10540948; doi:10.1371/journal.pone.0287097)
Supplement: Attachment — Submitted filename: PLos_one_Feb_23.docx [file pone.0287097.s004.docx]

**General Comments:**

Overall, I think this is a very informative study that adds to the current understanding of how parasites can alter crustaceans behavior and physiology, specifically, and the potential ecological and economic ramifications of parasites, at large. While I think my comments can greatly aid in the readability of this document, they are admittedly all relatively minor, as I think the authors work stands on its own.

**Abstract**

- In line 30, change “their” to the hosts’
- Split lines 34 – 38 into 2 sentences, one about the physiological parameters studied and one about the behavioral assays conducted
- Line 45- boldness and aggression are specific behavioral personalities that can be shown with repeated testing. It seems strange to include those terms here considering this paper isn’t about personalities
- Move the sentence in lines 44 – 45 up to right before the results
- Lines 44 – 47 don’t seem to fit in the abstract, or if the author feels they must be included, should be moved up to the methods part of the abstract instead of as the last sentence

**Introduction:**

- In general, the flow of the introduction seems a bit off. It immediately gives information about the host and parasite then go back to talking about parasite infection at large. All of the information is good, it’s just the order. Also, including transition sentences as the last sentence in each paragraph would greatly aid in flow for the reader
- Add a sentence at the end of the first paragraph to also include behavioral impact of parasites on hosts because this study includes physiological and behavioral impacts
- I found some studies (e.g. Gnanalingam and Bulter 2018) that say *P. argus* also eat gastropods, which could be important for parasite transmission, so should be included in their list of prey items in line 73
- Lines 75-78 don’t seem relevant to the paper, unless the parasite can be transmitted between 2 lobsters, which seems very unlikely

**Materials and Methods:**

- Lines 114-116, depth of tanks?
- Line 117- is there a known range for salinity and temperature during the time of the experiments that could be included here?
- Lines 124-126, include Ns for each
- Line 128, what is the N?
- Line 136 – 137- how were the cysts quantified?
- This response is called a tail flip in other crustaceans. Is it called that in lobsters?
- Were the lobsters used in these experiments fully intact?
- Lines 175 – 182 read like introduction material, not materials and methods, and should be moved accordingly. The information is pertinent to the paper
- Lines 212-214- the hepatopancreas also acts as a filter, removing toxins from the hemolymph
- Lines 218-227 also read as introduction material and should be moved
- Line 241 mentions these categories rendered samples of similar size. Does this mean the lobsters were of similar size or there was a similar N for all categories?
- No normality results are reported, so why was a Kruskal-Wallis test used?
- If lobsters were all dissected and metacercariae counted post-mortem anyway, why not just use these data for all analyses instead of using the visual tests of alive lobsters at all?

**Results:**

- While I have no doubt that the statistical analyses showed significant results for some of the escape response, figure 2 looks like there are no differences. Would bar graphs with error bars be more informative here?
- Same comment for figure 3

**Discussion**

- Lots of specific anatomical terminology is used, especially in lines 396-409. This terminology is definitely relevant to the findings, but adding another figure that is a labeled diagram of a lobster would greatly aid in readers’ understanding, especially if not familiar with crustacean anatomy
- Adding transition sentences throughout the discussion would also aid in flow, similar to the introduction
- Did that authors relate the fiddler crab behavior with 5-HT concentration for the example mentioned in 427-431? If so, that should be mentioned here because as written, this seems like 2 unrelated findings
- Line 439- *Orconectes virilis* has been reclassified as *Faxonius virilis*
