# Supplementary material for: Effects of Cymatocarpus solearis (Trematoda: Brachycoeliidae) on its second intermediate host, the Caribbean spiny lobster Panulirus argus
Source: PLoS One. 2023 Sep 29;18(9):e0287097. doi: 10.1371/journal.pone.0287097 (PMC10540948; doi:10.1371/journal.pone.0287097)
Supplement: Attachment — Submitted filename: Responses to reviewers.docx [file pone.0287097.s005.docx]

**Manuscript Number:** PONE-D-23-02958
**Manuscript Title:** Effects of *Cymatocarpus solearis* (Trematoda: Brachycoeliidae) on its second intermediate host, the Caribbean spiny lobster *Panulirus argus*

**Dear Editor: Our responses to reviewers’ comments are in blue.**

**Journal Requirements:**

When submitting your revision, we need you to address these additional requirements.

1. Please ensure that your manuscript meets PLOS ONE's style requirements, including those for file naming. The PLOS ONE style templates can be found at 
<https://journals.plos.org/plosone/s/file?id=wjVg/PLOSOne_formatting_sample_main_body.pdf> and 
<https://journals.plos.org/plosone/s/file?id=ba62/PLOSOne_formatting_sample_title_authors_affiliations.pdf>

2. We note that the grant information you provided in the ‘Funding Information’ and ‘Financial Disclosure’ sections do not match.

When you resubmit, please ensure that you provide the correct grant numbers for the awards you received for your study in the ‘Funding Information’ section.

R: Grant number has been corrected.

3. Please include captions for your Supporting Information files at the end of your manuscript, and update any in-text citations to match accordingly. Please see our Supporting Information guidelines for more information: <http://journals.plos.org/plosone/s/supporting-information>.

**R. Caption for Supporting Information has been added at the end of the manuscript. In-text citation matches (Line 249 of revised manuscript).**

**Reviewer 1.**

Digenean trematode life cycles are fascinating and wonderfully complex. Many interesting ecological questions can be tested at the intermediate host-stage, as this is where behavioral manipulation of hosts is theorized to occur. In this paper, the authors quantified the effects of *C. solearis* infection on the Caribbean spiny lobster *P. argus*, a second-intermediate host for the parasite. There were clear differences in biomarkers of infection (e.g., concentrations of glucose and serotonin) and physical performance indicators (e.g., escape response) as a function of infection intensity in *P. argus*, findings that increase the likelihood of trophic transmission to an appropriate definitive host.

I believe the manuscript meets all PLOS ONE publication criteria. The work is scientifically sound, and I have no concerns about the experimental design. However, I would suggest that the authors implement some additional statistical tests to take full advantage of the data that they have painstakingly collected. For the most part, the authors limited themselves to using non-parametric hypothesis tests (Kruskal-Wallis) to test for significant differences. These tests can be useful, but they must be interpreted carefully because of problems with statistical vs. biological significance (i.e., Johnson 1995). More to the point, these tests have no real predictive abilities. Given the wealth of data collected for this project, I think the manuscript could be improved using glms and/or mixed models. These analyses would address the question: which variable(s) account for most of the variation in infection intensity? As a reader, I was left wondering about this. I have more detailed suggestions along these lines in the Methods/Results section of this review.

- Johnson, D. H. (1995). Statistical sirens: the allure of nonparametrics. *Ecology*, *76*(6), 1998-2000.

R1: We appreciate the suggestion of using parametric analyses for the data. We have now used general linear models (GLM), i.e., conventional linear regression models for a continuous response variable given continuous and/or categorical predictors. We were worried about violating some assumptions of parametric tests, but this was not an issue since we used robust tests. Please see our response no. 7 (R7) below.

**Introduction**

Lines 79-89 -- why is visual assessment important? I think some additional context here would help. Is it just for researchers interested in the presence of the parasite, or do fishermen care as well? If appearance is important, this may be something to return to in the Discussion (beginning around line 460).

R2: If visual assessment of infection prevalence provided a good approximation to real prevalence, it would be a rapid, non-invasive way of assessing infection in the lobster populations, which would be of interest to both researchers and fishers. However, here we prove that visual assessment misses many infected lobsters and hence cannot be used for rapid assessments.

Lines 89-90 – the transition to the next paragraph about trophic transmission seemed a little abrupt. Perhaps another sentence is needed here?

R3. The Introduction has been completely rearranged to improve transition between paragraphs.

Lines 98-102 – The ideas here seemed a little vague. I think the reader may be left wondering why these parasites have more consequences for intermediate hosts. The intermediate host needs to be eaten for the parasite to complete its lifecycle in a definitive host. Perhaps this should be stated? Also, how would parasites affect the lobster fishery?

R4. Thank you for this suggestion. The paragraph has been revised.

**Methods/Results**

Line 182 – consider citing (and perhaps later discussing?) Shaw et al. 2009:

- Shaw, J. C., Korzan, W. J., Carpenter, R. E., Kuris, A. M., Lafferty, K. D., Summers, C. H., & Øverli, Ø. (2009). Parasite manipulation of brain monoamines in California killifish (Fundulus parvipinnis) by the trematode Euhaplorchis californiensis. *Proceedings of the Royal Society B: Biological Sciences*, *276* (1659), 1137-1146.

R5: Thanks for recommending this paper, which is relevant to our manuscript.

Suggestions for additional post-hoc testing:

It is possible to have a low p-value but a small effect size. In other words, the effect detected by the test that seemed so important (i.e., with the low p-value) is actually quite small. Cumming (2012) gives an excellent overview of this issue. To provide some additional context for your results from the Kruskal-Wallis tests, I might suggest testing the magnitude of effect for each significant p-value. The package “rstatix” is quite useful for basic statistical tests – I think Cohen’s D is preferred for parametric data and Wilcoxon effect size tests for non-parametric data.

# Cumming G. 2012. Understanding The New Statistics: Effect Sizes, Confidence Intervals, and Meta-Analysis (Multivariate Applications Series)

R6: After checking major assumptions for parametric tests, we have changed all statistical analyses to GLMs and do not use Kruskal Wallis tests in the revised manuscript.

Suggestions for model building using information theoretic approaches:

You probably have your own methods, but in the interests of being as helpful as possible I’ll share with you what packages I typically use for multimodel inference in R.

- glmmTMB – package for building and testing generalized linear mixed models that has a great suite of diagnostic tools for assessing model fit:

[https://cran.r-project.org/web/packages/glmmTMB/index.html](about:blank)

- DHARMa – add-on package with helpful tests for assisting with model convergence issues:

[https://cran.r-project.org/web/packages/DHARMa/vignettes/DHARMa.html](about:blank)

- AICcmodavg – package for ranking and comparing models using information theoretic approaches:

[https://cran.r-project.org/web/packages/AICcmodavg/index.html](about:blank)

You could bin the data into infected (1 or more cysts) and uninfected (0 cysts) categories. The goal here would be to rank and compare models testing the likelihood of infection. What variable(s) best predict whether a lobster is infected or not? Your response variable would be infection status (yes or no), a binary outcome (1 or 0), and you would fit the data to a binomial error distribution. In models that incorporate swimming performance as a predictor, you could consider adding trial number as a random effect as this might account for some of the variation in your response variable but would not be of biological/ecological importance.

You could also build models using infection intensity as your response variable (0 cysts; 1-10 cysts; 11-30 cysts; > 30 cysts). What variable(s) predict the likelihood of infection intensity in each case? You would fit the data to a negative binomial distribution if they showed evidence of overdispersion, which may be the case for the final category – otherwise, a Poisson would probably fit the data better. For variables that had multiple trials, consider using trial number as a random effect in your model structure.

R7. Thank you for all this interesting and very useful information. However, in our study we were not trying to find variables predicting whether a lobster was infected or not, or using infection intensity as the response variable. Quite the opposite: we were trying to find how infection intensity (the predictor) affected a number of response variables of the lobsters (i.e., components of the escape response, growth rate, concentration of metabolites or biogenic amines, etc.). Originally, we had planned on measuring the entire group of variables in the same lobsters, but this was not possible due to time constraints, logistic problems, death of several lobsters, molting of lobsters, incompatibility of some experiments, recording errors, etc. However, it is true that parametric tests are more powerful, and many are robust to departure from some assumptions. Therefore, following your advice, we have now changed all statistical analyses. We used multivariate analyses of variance (MANOVA) with a general lineal model (GLM) approach to test for potential effects of parasite intensity (with four levels: uninfected, lightly infected, moderately infected, and heavily infected) on two groups of variables that were measured in the same lobsters: (a) the components of the escape response, and (b) the concentration of metabolites in hemolymph. In (b), all MANOVA assumptions were met, but in (a), the assumption of homogeneity of the variance-covariance matrix was not met. However, MANOVA is robust to departure from this assumption when sample sizes are equal or nearly equal (which was the case) (Tabacknik & Fidell 2012; Nimon 2012). The rest of the variables (concentration of dopamine, concentration of serotonin, hemocyte count, hepatosomatic index, and growth rate) were individually subjected to ANCOVAs, using size as a covariate.

Upon doing the MANOVA for the components of the escape response, I noticed an unfortunate mistake in the calculation of acceleration (m/s/s) in the group of uninfected lobsters due to the use of an erroneous column in the Excell sheet. The spurious results had led us to conclude that there were significant differences in acceleration (and in work performed and force exerted, which are calculated from acceleration) of uninfected lobsters relative to the three groups of infected lobsters, when in fact the differences were not significant. In view of this mistake, we double-checked again all data, and everything else was correct. Thus, although acceleration did not differ among lobster groups, these groups did differ significantly in the concentration of serotonin and glucose in the hemolymph.

Tabachnick BG, Fidell LS (2012) *Using Multivariate Statistics, 6th edn*. Pearson, London.

Nimon KF (2012) Statistical assumptions of substantive analyses across the general linear model: a mini-review. *Front. Psychol.* 3: 322.

Some thoughts on lobster size as a predictor variable:

I think you could make an argument for using lobster size as a fixed effect (predictor) in your models, or as a random effect since there doesn’t seem to be a clear linear relationship between size and infection status (lines 280-281). Admittedly, this result is a little surprising since larger (older) organisms tend to have more cysts, which you mention (lines 248-249). However, it may also be possible for crustaceans to “clear” their cysts over time. Older cysts can appear brown and withered (Blakeslee et al. 2020), a result of the long-term crustacean innate immune response (Lee and Soderhall 2002); however, you did not find evidence of melanization (line 472). One other intriguing possibility for this non-linear relationship is that larger adults are foraging in different areas compared to smaller individuals and so are not in proximity as often to the first-intermediate host. I have some unpublished data from stone crabs *Menippe mercanaria* that support this idea. Smaller individuals (up to approx. 40 mm carapace width) are sympatric with Panopeid mudcrabs in shallow water oyster reefs. These individuals often have trematode cysts. Larger stone crabs, on the other hand, tend to burrow more and seek out deeper habitats, meaning that they are not as close on average to the first-intermediate host, the mudsnail *Ilyanassa obsoleta.* I rarely find cysts in these larger stone crabs.

All in all, since the biological relationship between size and infection status seems unclear, I might argue for using size as a random effect since it is not an important predictor of infection but may still account for some variation in your response variables.

- Blakeslee, A., Ruocchio, M., & Moore, C. S. (2020). Altered susceptibility to trematode infection in native versus introduced populations of the European green crab.
- Lee, S. Y., & Söderhäll, K. (2002). Early events in crustacean innate immunity. *Fish & shellfish immunology*, *12*(5), 421-437.

R8. In lines 249-249 of previous ms we were citing Koehler & Poulin (2010); ours is the first estimation of number of *C. solearis* cysts in *P. argus* lobsters. We found no relationship between lobster size and number of cysts by full dissection, so a larger number of cysts in larger organisms does not appear to apply to this system. Regardless, we included size as a covariate in the GLMs that we performed for the variables that were measured independently. We did this because most statistics books concur in that continuous variables such as size cannot be used as random effects but as covariates.

**Discussion**

I’ll mostly hold off on commenting on this section for now, as your results may change if you decide to incorporate my suggestions for additional analyses. Ultimately, I think what you have is great, but I think you could do a lot more with your data.

R9. Thank you very much for all your comments. The Discussion has indeed changed, mostly because of the detected mistake in the estimation of acceleration (and hence in force and work, which are derived from acceleration) for uninfected lobsters. Upon correcting the data, we found no significant effect of parasite intensity on any component of the escape response, and the Discussion was changed accordingly.

One note on the serotonin (5-HT) results:

Would elevated levels of 5-HT affect lobsters’ ability, or interest, to shelter together in casitas? How would this affect the fishery?

R10. It might, since in other decapods higher levels of 5-HT increase their “will to fight”. But whether this would affect the fishery remains to be determined. Therefore, we modified the Discussion like this (new Lines 464-469): “the higher concentration of 5-HT in moderately and heavily infected lobsters may cause these lobsters to become more aggressive and attempt to fight an attacker beyond their capabilities, potentially affecting the response of lobsters in predator-prey encounters. It may also affect the social behavior of lobsters, with unknown consequences for casita-based fisheries. A comparison of defense and antipredator behaviors between uninfected and infected lobsters should be conducted in future studies.”

**WE THANK REVIEWER 1 FOR THE THOROUGH REVIEW AND HELPFUL COMMENTS.**

**Reviewer 2**

**General Comments:**

Overall, I think this is a very informative study that adds to the current understanding of how parasites can alter crustaceans behavior and physiology, specifically, and the potential ecological and economic ramifications of parasites, at large. While I think my comments can greatly aid in the readability of this document, they are admittedly all relatively minor, as I think the authors work stands on its own.

R. We appreciate the positive feedback.

**Abstract**

- In line 30, change “their” to the hosts’

R. Done.

- Split lines 34 – 38 into 2 sentences, one about the physiological parameters studied and one about the behavioral assays conducted

R. Done.

- Line 45- boldness and aggression are specific behavioral personalities that can be shown with repeated testing. It seems strange to include those terms here considering this paper isn’t about personalities

R. The terms bolder and more aggressive have been deleted from the Abstract.

- Move the sentence in lines 44 – 45 up to right before the results

R. Done.

- Lines 44 – 47 don’t seem to fit in the abstract, or if the author feels they must be included, should be moved up to the methods part of the abstract instead of as the last sentence.

R. These lines were removed from the Abstract.

**Introduction:**

- In general, the flow of the introduction seems a bit off. It immediately gives information about the host and parasite then go back to talking about parasite infection at large. All of the information is good, it’s just the order. Also, including transition sentences as the last sentence in each paragraph would greatly aid in flow for the reader

R. Thank you for your suggestions. The Introduction has been extensively rearranged.

- Add a sentence at the end of the first paragraph to also include behavioral impact of parasites on hosts because this study includes physiological and behavioral impacts

R. Thanks for the suggestion.

- I found some studies (e.g. Gnanalingam and Bulter 2018) that say *P. argus* also eat gastropods, which could be important for parasite transmission, so should be included in their list of prey items in line 73

R. The diet of these lobsters is opportunistic and very diverse. They eat bivalves, gastropods, chitons (i.e. mollusks), cirripedes and all kinds of decapods, (crustaceans), ophiurids, urchins, sea cucumbers (echinoderms) and all kinds of annelids (worms). Because of this and because there is no trophic transmission of these trematodes from the first to the second intermediate host, we only mentioned the higher taxa.

- Lines 75-78 don’t seem relevant to the paper, unless the parasite can be transmitted between 2 lobsters, which seems very unlikely

R. These lines were intended to explain why casitas are used in some fisheries, such as the one from which we obtained the lobsters, and because, as suggested by Reviewer 1, the increase in serotonin in infected lobsters may alter their social behavior, which is now addressed in the Discussion.

**Materials and Methods:**

- Lines 114-116, depth of tanks?

R. Depth of tanks has been added.

- Line 117- is there a known range for salinity and temperature during the time of the experiments that could be included here?

R. Yes. The ranges have been added.

- Lines 124-126, include Ns for each

R. Done.

- Line 128, what is the N?

R. The sample size has been added.

- Line 136 – 137- how were the cysts quantified?

R. All cysts found in a lobster upon full dissection were counted.

- This response is called a tail flip in other crustaceans. Is it called that in lobsters?

R. Yes. This name has been added.

- Were the lobsters used in these experiments fully intact?

R. Yes, except for 4, which were lacking 1 appendix (leg or antenna), and 2 which lost it during the experiment.

- Lines 175 – 182 read like introduction material, not materials and methods, and should be moved accordingly. The information is pertinent to the paper

R. These lines were added here as an explanation of why we considered important to compare that characteristic. However, following your advice, we have moved a shortened version of all this material (and similar parts related to other characteristics) to the last part of the Introduction.

- Lines 212-214- the hepatopancreas also acts as a filter, removing toxins from the hemolymph

R. We have removed this piece of information.

- Lines 218-227 also read as introduction material and should be moved.

R. Same response as to Lines 175-182.

- Line 241 mentions these categories rendered samples of similar size. Does this mean the lobsters were of similar size or there was a similar N for all categories?

R. It means a similar N for all lobster groups. Sentence has been rephrased.

- No normality results are reported, so why was a Kruskal-Wallis test used?

R. We have changed all the statistical analyses to parametric tests. We now used MANOVAs and ANOVAs with a General Lineal Model (GLM) approach.

- If lobsters were all dissected and metacercariae counted post-mortem anyway, why not just use these data for all analyses instead of using the visual tests of alive lobsters at all?

R. In previous studies, prevalence of infection by *C. solearis* in wild lobsters was estimated only by visual assessment. We wanted to test how “accurate” these visual assessments are, because they could be used to conduct rapid assessments elsewhere. Therefore, we conducted the validity measures but find out that visual assessment missed many infected lobsters, so it is not a good method for rapid assessments.

**Results:**

- While I have no doubt that the statistical analyses showed significant results for some of the escape response, figure 2 looks like there are no differences. Would bar graphs with error bars be more informative here?

R. These graphs have been changed because we found a mistake in the data processing, for which we apologize. Acceleration had been wrongly estimated (see response R7 to Reviewer 1). After correcting the mistake, no significant differences were found in any components of the escape response. Analyses and figures have now been corrected. However, we believe that, unlike column graphs with error bars, box plots show the actual distribution of the raw data, so they are a good choice when comparing groups.

- Same comment for figure 3.

R. Please see previous response.

**Discussion**

- Lots of specific anatomical terminology is used, especially in lines 396-409. This terminology is definitely relevant to the findings, but adding another figure that is a labeled diagram of a lobster would greatly aid in readers’ understanding, especially if not familiar with crustacean anatomy

R. Thanks for this suggestion. We have added a photo of a lobster with the relevant names (Fig. 1 in revised ms).

- Adding transition sentences throughout the discussion would also aid in flow, similar to the introduction

R. The discussion has been changed to address the amended results. Care has been taken to enhance the flow between paragraphs.

- Did that authors relate the fiddler crab behavior with 5-HT concentration for the example mentioned in 427-431? If so, that should be mentioned here because as written, this seems like 2 unrelated findings.

R. Yes, it was the same authors. The sentence has been rephrased to improve clarity.

- Line 439- *Orconectes virilis* has been reclassified as *Faxonius virilis*

R. Thanks for catching this. The name has been corrected.

**WE THANK REVIEWER 2 FOR THE THOROUGH REVIEW AND HELPFUL COMMENTS.**
