# Supplementary material for: Effects of Cymatocarpus solearis (Trematoda: Brachycoeliidae) on its second intermediate host, the Caribbean spiny lobster Panulirus argus
Source: PLoS One. 2023 Sep 29;18(9):e0287097. doi: 10.1371/journal.pone.0287097 (PMC10540948; doi:10.1371/journal.pone.0287097)
Supplement: Attachment — Submitted filename: revisions of PONE-D-23-02958.docx [file pone.0287097.s006.docx]

**Manuscript Number:** PONE-D-23-02958
**Manuscript Title:** Effects of *Cymatocarpus solearis* (Trematoda: Brachycoeliidae) on its second intermediate host, the Caribbean spiny lobster *Panulirus argus*

Response to authors’ revisions:

I have read the authors’ response-to-reviewers document and the revised manuscript, which overall is much improved. The authors have done a thorough job with their revisions. In the process of reanalyzing the data, they uncovered and corrected a typo in the original dataset that would have affected their results and discussion. They also switched their primary method of analysis from hypothesis testing (i.e., Kruskal-Wallis) to regression-based methods, which are more comprehensive and informative. Parts of the Introduction and Discussion were reorganized as needed based on feedback from both reviewers.

At this point, I have only minor requests for clarification, primarily with how the stats are reported. I am trying to be as helpful as possible, as I think the work is interesting and very informative!

- Methods

You mention that you used general linear models (line 314) as opposed to generalized linear models. The former assumes that the residuals from your dependent variable are normally distributed while the latter does not. Parasite count data is often non-normal…I believe your dependent variable was parasite intensity? (line 315). I think it would help to clarify that the residuals of your data were normally distributed and then report that prior to discussing how you used GLMs. Otherwise, you should switch to using generalized linear models, which are preferred for modeling data with non-normal residuals.

If you stick with the general linear models, I might suggest reporting a few other test statistics for your ANCOVA results. By themselves, p-values convey very little information about the magnitude of effect, i.e., how powerful, or significant, your results are. I would suggest including information on marginal means and their associated 95% confidence intervals and reporting this for each significant effect in your results. Here’s some additional information on reporting CIs and marginal means for ANCOVAs: <https://www.medcalc.org/manual/analysis-of-covariance.php>

- Other

I think it would help to have a final concluding sentence in your Abstract that kind of ties things together, a more general statement about how your results apply more broadly. You have something like this in your conclusion section (p. 29) around lines 626-629.
